# Eye state asymmetry during aquatic unihemispheric slow wave sleep in northern fur seals (*Callorhinus ursinus*)

**DOI:** 10.1371/journal.pone.0217025

**Published:** 2019-05-22

**Authors:** Jessica M. Kendall-Bar, Alexei L. Vyssotski, Lev M. Mukhametov, Jerome M. Siegel, Oleg I. Lyamin

**Affiliations:** 1 Department of Ecology and Evolutionary Biology, University of California Santa Cruz, Santa Cruz, CA, United States of America; 2 Institute of Neuroinformatics, University of Zurich / Swiss Federal Institute of Technology (ETH), Zurich, Switzerland; 3 A.N. Severtsov Institute of Ecology and Evolution, RAS, Moscow, Russia; 4 Utrish Dolphinarium– 84 Ltd., Moscow, Russia; 5 Department of Psychiatry, University of California Los Angeles, Los Angeles, CA, United States of America; University of Oxford, UNITED KINGDOM

## Abstract

Unihemispheric slow wave sleep (USWS) is a unique form of sleep in which one brain hemisphere maintains low voltage electrical activity indicative of waking while the opposite exhibits slow wave electrical activity indicative of sleep. USWS is present in several marine mammals and in some species of birds. One proposed biological function of USWS is to enable the animal to monitor the environment to detect predators or conspecifics. While asymmetrical eye state was often observed during behavioral sleep in birds and marine mammals, electrophysiological (electroencephalogram, EEG) correlates between the asymmetry of eye state and EEG of two cortical hemispheres have not been reliably established. This study examined the association between eye state and EEG activity during aquatic sleep in two subadult northern fur seals (*Callorhinus ursinus)*, taking advantage of the simultaneous visibility of both eyes when the seals were in the prone position. We found that during USWS the eye contralateral to the sleeping hemisphere was closed on average 99.4±0.1% of the recording time. The eye contralateral to the waking hemisphere opened briefly for on average 1.9±0.1 sec with a rate of 8.2±1.0 per min. This eye was open on average 24.8±2.5% of the USWS time and it was closed no longer than 3 sec, on average 39.4±5.6% of the time. These data indicate that fur seals sleep in seawater by having intermittent visual monitoring. Our findings document the extent of visual monitoring of both eyes during USWS and support the idea that USWS allows intermittent visual vigilance. Thus, USWS serves two functions in the fur seal, facilitating movement and visual vigilance, which may also be the case in cetaceans.

## Introduction

While terrestrial mammals and humans display bilaterally symmetrical synchronized electroencephalogram (EEG) activity during slow-wave sleep (SWS), cetaceans and some pinnipeds exhibit unihemispheric slow wave sleep (USWS), where one hemisphere is “awake”, displaying low voltage EEG, and the other is “asleep”, displaying high voltage EEG slow-wave activity [1–3]. It has been suggested that USWS enables 1) visual vigilance via continued monitoring of the marine environment to detect predators and maintain pod coherence [2–4], and 2) movement to prevent water aspiration when breathing [1] and aids in thermoregulation [3,5]. The available cetacean data on unilateral eye closure during USWS support its sentinel function [2,6]. With regards to the second hypothesis, USWS seems to play a role in maintaining motion and thermoregulation in cetaceans, since they can swim and generate heat via muscle activity during USWS [1,3].

Unlike cetaceans, pinnipeds can sleep both on land and in water. When sleeping on land, pinnipeds are motionless. They predominantly display SWS in two hemispheres (bilateral SWS) as seen in land mammals. However, fur seals (the family Otariidae) can also exhibit highly asymmetrical SWS on land, with pronounced differences between the amplitude of EEG slow waves in the two cortical hemispheres [6–10]. This type of SWS resembles the USWS in cetaceans [1–3].

**Fig 1:**
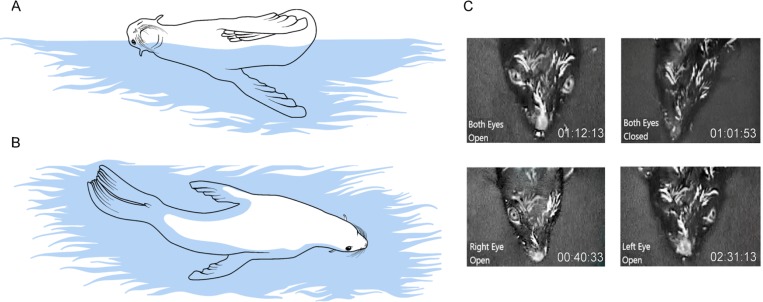
Sleep postures and eye states in fur seals in water. Fur seals sleep in water at the surface in the lateral and prone positions. (A) During USWS in the lateral position, the “waking” hemisphere is contralateral to the moving flipper, while the “sleeping” hemisphere is ipsilateral to the moving flipper. When in this position, the fur seal’s eye which is directed to the sky (contralateral to the sleeping hemisphere) is often closed, while the eye facing the water (contralateral to the waking hemisphere) is usually not visible. (B) When the fur seal sleeps in the prone position, both eyes are visible most of the time and the motion is minimal. (C) Symmetrical and asymmetrical eye state in a fur seal resting in water in a prone position.

When in seawater, fur seals predominantly display USWS [7,10]. They usually sleep at the surface in a distinct, asymmetrical posture with both hind flippers and one fore flipper above the surface [10–12]. The other front flipper paddles to stabilize the posture (Fig 1A). In this lateral position, the “waking” hemisphere (the one with low voltage EEG) is contralateral to the moving flipper, while the “sleeping” hemisphere (the one with high voltage EEG) is always ipsilateral to the moving flipper. The correlation between the patterns of EEG and movement in unihemispherically sleeping fur seals provides strong evidence that USWS enables motion of the fur seal in water [7,10].

On the other hand, the sentinel / antipredator vigilance function of USWS has not yet been investigated in pinnipeds. In our prior study we briefly examined eye state during terrestrial sleep in fur seals [6]. However, these land-based data are not entirely representative of the cognitive demands of sleep in the wild, where fur seals are under pressure from aquatic predators, such as killer whales and great white sharks [12,13]. In addition, in our study [6] we documented the state of just one eye.

Sleep with one eye open appears to be widespread among birds [14]. Some birds also display interhemispheric EEG asymmetry during SWS, although the degree of the asymmetry is less pronounced (e.g., can be visually undetectable as in the pigeon; [15,16]) than during USWS in marine mammals. It was reported that, in at least 5 species, the hemisphere contralateral to the closed eye exhibited EEG slow waves typical of SWS with both eyes closed, and the hemisphere contralateral to the open eye exhibited intermediate EEG, between SWS and wakefulness (e.g., [17–19]). However, the degree of such a correlation was measured and quantitatively evaluated in only a few studies (e.g., in the pigeon and in the mallard duck in the laboratory and indirectly in the great frigatebirds during flight; [16,20,21]).

In summary, the available experimental data appear to support the hypotheses that USWS serves a sentinel function in cetaceans and in birds. At the same time, another set of data convincingly indicates that USWS allows motion (movement of one flipper to stabilize the sleep posture) in unihemispherically sleeping fur seals. It is not clear whether USWS serves a single or multiple functions in different groups of marine mammals and birds.

The quantification of eye state in marine mammals during sleep in the water has traditionally been very difficult. In case of the fur seal sleep, the typical aquatic sleep posture is a lateral position, where the fur seal’s eye that is contralateral to the awake hemisphere is usually obstructed by the water it faces (Fig 1A). This study makes use of a prone posture in which both eyes are visible (Fig 1B and 1C) while motion is minimal (virtually absent). The data we collected provide further evidence of the association between eye state and brain activity in the fur seal to better understand the function(s) and biological role of USWS.

## Materials and methods

### Animals

The data were collected from 4 subadult northern fur seals (*Callorhinus ursinus*). The animals (3 males and 1 female, 18–25 kg, 2–5 years old) were captured in the Commander Islands (5.28548236°N, 165.75603247°E) 1 or 2 years before the study. The experiments were conducted at the Utrish Marine Station of the A.N. Severtsov Institute of Ecology and Evolution of the Russian Academy of Sciences (Black Sea, Russia; 44.70493445°N, 37.47129142°E). All procedures were approved by the Veterans Affairs Greater Los Angeles Healthcare System and the A.N. Severtsov Institute. The capture permits were issued by the Russian Federation Federal Agency for Fishery.

### Experimental design

The fur seals were housed individually in an indoor 4x4x1.8 m pool filled with seawater. The experiments were designed to examine characteristics of REM sleep in fur seals in seawater [10].

All fur seals were implanted under general anesthesia (isoflurane, 1–3%) with two pairs of stainless-steel screws (1 mm in diameter) epidurally for EEG recording from symmetrical fronto-occipital or fronto-parietal derivations of the right and left hemispheres. A pair of screws was also implanted in the supraorbital bone above the eyes to record the electrooculogram. Four Teflon-coated multi-stranded stainless-steel wires (0.3 mm in diameter) were inserted into the nuchal muscles to record electromyogram activity. Two longer wires were implanted subcutaneously behind the fore-flippers to record electrocardiogram activity. The leads were soldered to a micro-connector and attached to the skull with acrylic cement. After implantation, the seals were returned to the pool. They were allowed at least 5 days to recover before the experiments started. During this time, the seals were given antibiotics and analgesics via ingested fish. By the second day after surgery, the implanted seals resumed eating fish and appeared to be in good condition. This surgical procedure has been employed in several prior studies (e.g., [6–10]).

Five to seven days after the surgery a micro-plug on the fur seal head was connected to a datalogger (Neurologger, bandwidth 1–115 Hz, acquisition rate 200 Hz, [22]) via a flexible 45 cm long, low noise coaxial cable. The datalogger allowed continuous recording of all the parameters for 5 days. The datalogger was attached to a platform (6x10x0.5 cm) on the seal’s back using cable ties. The platform was made from a neoprene resin and glued to the seal’s fur with a neoprene adhesive (McNett, WA) during the surgery. This type of datalogger has also been used to study sleep in birds [21] and marine mammals [3,10,23].

After the fur seal was instrumented with a datalogger, the animal was allowed 2–3 days to habituate to the recording equipment. During these days, the seals were given access to a low level of seawater (10 cm). They also had access to a dry wooden platform positioned above the water. Under these conditions, the seals rested and slept while lying on the platform. After 2–3 days, the platform was removed, and the pool was filled with seawater to a level of 1.2 m starting the “in seawater” period. The seals were in seawater for 10–14 days.

Every 5 days, the data were downloaded from the datalogger and converted to Spike2 Software format (Cambridge Electronic Design, UK) for visual and spectral power analysis. In order to do this, the seal was restrained for about 5 min when the pool was completely drained. The seals were accustomed to this short procedure. The seals’ behavior and eye state (whenever the eyes were visible) were continuously video recorded with 4 high-resolution remote-control cameras fitted with infrared light sources.

The seawater in the pool was changed daily between 08:00 and 08:30. The seals were fed fish twice a day and kept on a 12h light (400 lux; onset at 08:00) and 12h dim light (30 lux; onset at 20:00) cycle. The average air temperature in the pool (above the water) varied between 18–25°C. The average water temperature varied between 10–25°C (corresponding to that in the Black Sea). However, for each individual seal the daily variation of the air temperature did not exceed 5°C and the daily variation of the seawater temperature did not exceed 3°C.

### Data analysis

Rest, as is further described below, was quantified on 45 out of the total 46 days spent in seawater for all 4 fur seals (S1 Table). For 32 of those 45 days, we were able to score and quantify SWS for all 24 hours. On the remaining days, reliable scoring was not possible due to artifacts in the EEG of either one or both hemispheres. These data losses were caused by recording wire damage or data logger failure, [10]). All 4 seals (A-D, S1 Table) rested in the lateral position. Two seals (A and B) also rested in a prone position.

The behavior of fur seals in seawater and the polygrams (records of the EEG of two cortical hemispheres, electromyogram, electrooculogram and electrocardiogram) when in seawater was scored visually in 20-sec epochs into several sleep and wakefulness stages, including active waking (AW), quiet waking (QW), left or right asymmetrical SWS, and rapid eye movement (REM) sleep based on the criteria used in prior studies [6–10].

AW in fur seals in seawater included swimming, grooming, and feeding. QW was scored when the animal rested at the surface, looked around, slowly groomed, or became motionless, as long as low voltage desynchronized EEG was recorded in both cortical hemispheres.

SWS occurred in seawater at the surface in two characteristic postures: the lateral and prone positions (Fig 1A and 1B). Most of the time, the seal’s head was above the surface and its nostrils were in the air. USWS was scored when EEG slow waves at least 2-fold above the waking low voltage desynchronized EEG activity occurred in one (“sleeping”) hemisphere for at least of 50% of the epoch time simultaneously with the waking EEG pattern in the other (“waking”) hemisphere. Asymmetrical SWS was scored when low voltage EEG slow waves (amplitude at least 2-fold above the waking EEG activity, occupying at least 50% of the epoch time) were recorded in one hemisphere in parallel with EEG slow waves of higher amplitude (also occupying at least 50% of the epoch) in the other. When in seawater, the majority of visually detected asymmetrical SWS in fur seals was characterized by highly pronounced interhemispheric EEG asymmetry resembling USWS in cetaceans. Those episodes will be referred to as USWS, R-USWS or L-USWS (USWS in the right and left hemispheres, respectively).

Short REM sleep episodes recorded in fur seals in water were characterized by the head sinking towards the water, eye and muscle jerks, body twitches, breathing, heart rate irregularity and release of air bubbles. They usually occurred in series and each episode was terminated by brief awakening. The seals opened both eyes and paddled more intensely to resume the sleep posture [10].

Rest was defined as a period of behavioral quiescence which included episodes of QW, SWS and REM sleep. It started when the seal took the lateral or prone sleep posture and ended when the seal resumed active behavior (swimming or grooming). Periods of rest alternated with longs periods of AW.

As we showed in a prior study [8], the interhemispheric EEG asymmetry during SWS in fur seals is maximally expressed in the range of 1.2–4.0 Hz. It is also present in other frequencies (e.g., 4.0–16.0 Hz) but it is less pronounced. Therefore, in this study we chose to characterize the intensity of SWS and the degree of interhemispheric EEG asymmetry by the power in the frequency range of 1.2–4.0 Hz (also called slow wave activity or SWA). It was computed in consecutive 5 sec epochs for each hemisphere by fast Fourier transformation using Spike 2 software. Epochs containing artifacts were excluded. After that the average power in each hemisphere was calculated for 20-sec epochs (that is in 4 consecutive 5 sec epochs). Then we standardized the EEG power (the range of 1.2–4.0 Hz) in each hemisphere in each 20-sec dividing by the average power of the same frequency range in the same hemisphere during the artifact-free QW epochs [8–10].

The state for both eyes was scored visually based on video records as open, closed or non-visible with one-second resolution. Usually fur seals opened their closed eye to full size instantly while they closed their open eye slowly. We scored the eye state as closed when the eyelids fully obstructed the pupil. Blinks were not counted. The duration of opening of each eye was then calculated per 20-sec epochs and matched with the EEG power (1.2–4.0 Hz) in the left and right hemispheres. The duration of episodes of opening and closure of each eye was counted as the number of seconds the eye was open or closed continuously.

The difference between the power spectra in the two hemispheres and state of two eyes was evaluated using the paired T-test and the Wilcoxon signed rank test. All comparisons passed the test for normality. The results of both treatments reached same conclusions while the level of significance estimated by the Wilcoxon test was slightly smaller. The results of the paired T-test are presented in the main text while the results of the Wilcoxon test (in comparison with the T-test) can be found in the supplementary data file (S1 File). The reported values are means ± SEM. All statistical analyses were performed using Sigma Plot 11.0 Software.

## Results

Over the period of this study, all 4 fur seals predominantly rested and slept in seawater in the lateral position (87±8% of total rest time, S1 Table) while 2 out of 4 seals (one male and one female, seal A and B, respectively) rested and slept in the prone position. The data from these two seals were used to examine the association between eye state and USWS as described below.

**Fig 2:**
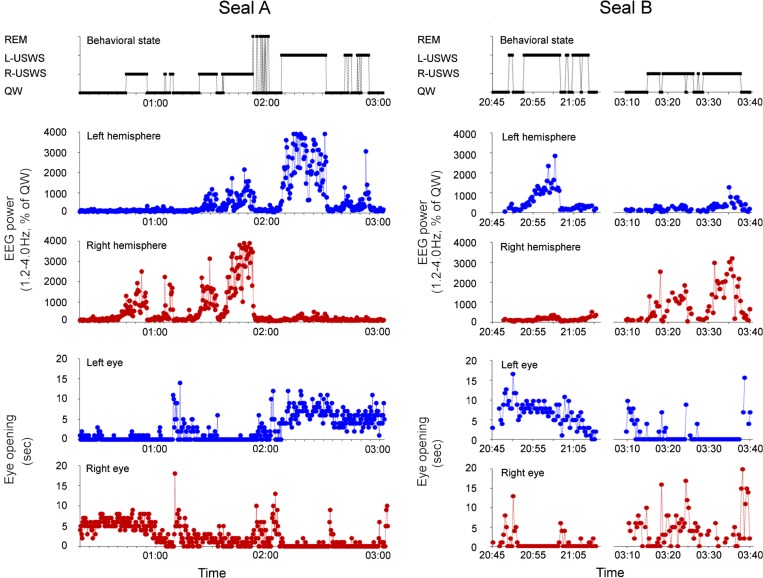
Association between EEG power and eye state in fur seals sleeping in seawater in a prone position. Diagrams show behavioral state scored as QW, R-USWS, L-USWS and REM sleep, then standardized EEG power in the range of 1.2–4.0 Hz in the left and right hemispheres (shown as percent of the average power in QW in the corresponding hemisphere) and the amount of time during which the left and right eye were open in corresponding 20-sec epochs in 2 fur seals (A and B) during sleep in a prone position. Several episodes of USWS in fur seal A occurred within one rest period first in the left and then in the right hemisphere. Two episodes of USWS in seal B (L-USWS and then R-USWS) were recorded for one night. Dotted lines connect markers representing data for consecutive 20-sec epochs. Several interruptions mark gaps when the data were not collected (due to artifacts in the EEG caused immersion of the head into the water or when the eyes were not visible).

**Fig 3:**
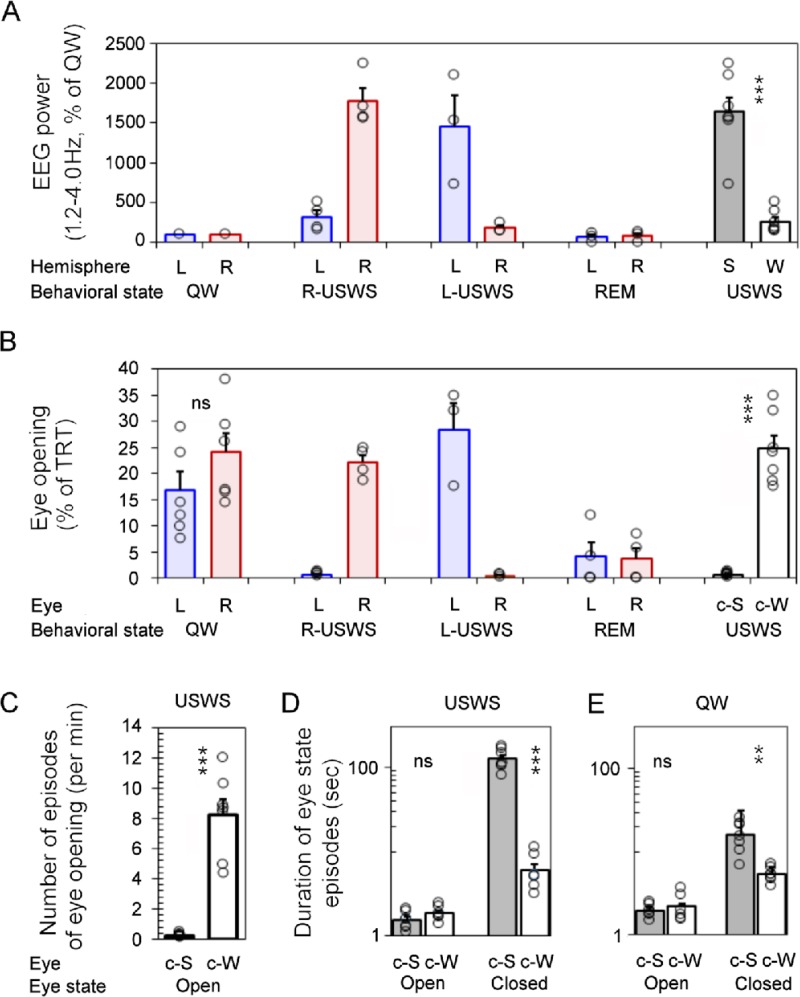
Association between behavioral state and eye state in fur seals in water. (A) Standardized EEG power in the range of 1.2–4.0 Hz in two cerebral hemispheres (shown as percent of the average power in QW in the corresponding hemisphere) during QW, R-USWS, L-USWS, USWS (R-USWS and L-USWS) and REM sleep in 2 fur seals. (B) Total amount of the open state of the left and right eye during waking and sleep states. (C-E) Number and duration of the episodes of eye opening and closure during USWS (C, D) and QW (E). S and W correspond to sleeping and waking hemispheres. L and R correspond to left and right hemispheres or eyes. c-S and c-W correspond to which eye is contralateral to the sleeping and waking hemisphere, respectively. During QW the eyes were classified as c-S or c-W based on the adjacent USWS. Open circles represent the means for individual USWS, QW and REM sleep episodes recorded in 2 seals (S2 Table). Bars are grand average (mean) ± SEM for all episodes of QW and USWS. The difference between the power and the state of the eye was tested for all 7 episodes of USWS and 7 adjusted episodes of QW recorded in seals A and B. **,*** Statistical significance (the paired T-test) with p<0.01 and <0.001, respectively. ns–difference was not significant (p>0.05; S1 File).

A total of 6 periods of rest (709 min or 11.8 h) occurred in 2 fur seals in a prone position (S2 Table). For all periods combined, USWS (Fig 2) occupied 53.3% of the total recording time (TRT). The average standardized slow wave EEG power (frequency of 1.2–4.0 Hz) ranged from 734 to 2254% of the average power during QW in the sleeping hemisphere and from 144 to 509% in the waking hemisphere (Fig 3A). The difference between the two hemispheres was highly significant (p<0.001, the paired t-test). QW made up most of the remainder of the rest periods (on average 45.2% of TRT) except for short periods of REM sleep (<1.5%, S2 Table). Episodes of REM sleep in water were relatively rare compared to their presence on land [10] but could sometimes be recorded after a period of several episodes of USWS (Fig 2). Individual REM sleep episodes lasted between 2 and 132 sec (on average 21±2 and 24±2 sec, respectively in seal A and B) and they occurred in series of 2 to 14 (on average 4.4±0.6 and 4.3±0.6 per series). Episodes of REM in series were interrupted by brief bilateral arousal, which met the criteria of QW. The last REM sleep episode in a series turned to longer periods of wakefulness or a new period of USWS. More details on the parameters of REM sleep in fur seals in water can be found in our recent publication [10].

For all rest episodes combined, the left, right, and both eyes were visible 90, 90 and 81% of TRT, respectively. As shown in Fig 2 and Fig 3A and 3B eye there was a clear association between the eye state and USWS in fur seals sleeping in the prone position. The eye contralateral to the sleeping hemisphere was mostly closed, accounting for between 99.0–99.7% of the time the eyes were visible (range for 7 episodes of USWS in 2 seals, on average 99.4±0.1%). The other eye, which was opposite to the waking hemisphere, was closed between 64.9–81.8% (75.2±2.5%) of the USWS time, which means it was open 18.2–35.1% (24.8±2.5%) of the time. This eye was usually closer to the water or submerged. The difference between the times that each of the two eyes was open during USWS was highly significant (p<0.001).

During USWS the fur seal eye contralateral to the waking hemisphere opened 4–12 times per minute (on average 8.2±1.0/min) while the eye contralateral to the sleeping hemisphere opened only once every 2–5 min (p<0.001, Fig 3C). The continuous opening of both eyes was short and lasted only several seconds. The average duration of the episodes of eye opening (contralateral to the waking or sleeping hemisphere) lasted less than 2 sec and did not differ (p>0.05, Fig 3D).

Unlike the openings, the duration of eye closure did differ during USWS (p<0.001). The eye contralateral to the waking hemisphere closed on averaged for 6.1±1.1 sec while the eye contralateral to the sleeping hemisphere could be closed during USWS for as long as 13 min (on average 126.2±13.2 sec) with 65–89% of the episodes being longer than 10 sec (Fig 3D). At the same time, between 4–23% of those episodes lasted only 1 sec, 9–54% less than 2 sec and 10–72% less than 3 sec. The eye contralateral to the waking hemisphere did not close for a period longer than 1 sec on average 27.0±2.9% of USWS, longer than 2 sec—32.5*±*4.4% and longer than 3 sec—39.2±5.6% of USWS (S3 Table).

The degree of EEG asymmetry in fur seals sleeping in water was associated with the degree of asymmetry in eye state. For example, if the degree of EEG asymmetry between the two hemispheres decreased (from USWS to asymmetrical SWS), so did the asymmetry of eye state (e.g. the episode of R-USWS in Seal A in Fig 2). Likewise, if EEG asymmetry stayed relatively constant, so did eye state (e.g., the episodes of L-USWS in both seals in Fig 2).

Eye state asymmetry in fur seals was also recorded during periods of QW. This asymmetry mirrored that which was observed during the adjacent (either preceding or following) period of USWS (Fig 2, the episode of QW before R-USWS in seal A and the episode before L-USWS in seal B). As in the adjacent period of USWS, during QW, the eye contralateral to the sleeping hemisphere was closed for more time than the eye which was contralateral to the waking hemisphere (p<0.01, Fig 3E). Both seals in the current study spent more time in R-USWS than in L-USWS (S2 Table). As a consequence, there was a slight overrepresentation of eye state asymmetry, manifested in a greater total amount of time the left eye was closed (Fig 3B) and consistent with a greater amount of R-USWS. The average duration of eye opening during QW did not differ from average eye opening duration during USWS (p>0.05, Fig 3E).

During REM sleep, both eyes were closed (88–100% of the time). They opened briefly during most intense eye jerks (on average less than 4% of the time, Fig 3B) but never as wide as during QW and USWS.

## Discussion

Unilateral eye opening / closure has been reported in many species of behaviorally sleeping birds (also called “monocular–unihemispheric” or Mo-Un sleep (reviewed in [14,19,20,24,25]) and to a lesser extent in cetaceans (reviewed in [3]) and in reptiles [26,27]. It is widely assumed that USWS serves a predator detection function referring to the evidence that the eye contralateral to the waking hemisphere remains open during USWS. Asymmetrical eye state is often used as a measure of USWS. However, electrophysiological (EEG) correlates of asymmetrical eye state in birds and marine mammals have not been well established and validated.

The most convincing evidence of the association between cortical EEG and eye asymmetry in birds came from the two studies performed in the sleeping mallard duck [20] and in the pigeon [16]. The studied ducks spent more time with one eye closed while at the edge of a group when the open eye was more often directed away from the center of the sleeping group. During SWS with interhemispheric EEG asymmetry, the open eye was more often contralateral to the hemisphere with low voltage EEG and the closed eye was contralateral to the hemisphere with higher voltage EEG. The interhemispheric EEG asymmetry in the pigeon was less expressed and not always detectable by visual inspection [15]. Nevertheless, when averaged across all episodes, the association between EEG power and unilateral eye state was detectable using the period amplitude spectral analysis [16]. Other bird studies generally reported a similar association between the asymmetry in eye state and EEG state of two cortical hemispheres without providing extensive experimental evidence (e.g., [17,18]).

The lack of experimental data on the association between eye state and cortical EEG asymmetry in birds can be explained by difficulties in detecting concurrent, instantaneous changes, and because vigilance can be maintained in different ways [24,25]. In addition to antipredator vigilance, episodes of Mo-Un sleep have been implicated in functions similar to that which are attributed to SWS in humans and land mammals such as recovery, learning and memory consolidation. For instance, in the domestic chick, the Mo-Un sleep pattern prevailed in the hemisphere that dominated during wakefulness [28]. Thus, in agreement with earlier studies in humans and rodents, episodes of Mo-Un sleep may allow more sleep to occur in the hemisphere which was more activated according to the “use-dependent paradigm” [29–31].

The previous marine mammal data on the association between USWS and asymmetrical eye opening / closure are based on the studies in two species of cetaceans (the beluga and bottlenose dolphin) and in two studies in semiaquatic pinnipeds (the walrus and northern fur seal). Each cetacean set of data was collected in a single individual housed in a small pool [2,6]. The pinniped data were collected in one captive young walrus [23] and in two subadult fur seals [6] sleeping on land in small enclosures. Only one of two eyes was visible the majority of the time. In all cases the studied cetaceans and pinnipeds were not free to move around due to the cables connecting the animals to the recording equipment.

The current findings are based on recording the state of two eyes and EEG of both cortical hemispheres during aquatic sleep when fur seals were free to swim around the pool without restriction. The prone sleep posture in the fur seal allowed us to examine and isolate the relationship between brain and eye activity while removing any effect due to motion and with the added benefit of being able to record data from both eyes simultaneously. Our study demonstrates that the asymmetry in the state of two eyes in the fur seal alternates in parallel with the state of two cerebral hemispheres without noticeable changes in the seal’s position or motion pattern. During USWS, the eye contralateral to the sleeping hemisphere is closed while the eye contralateral to the waking hemisphere periodically opens. This is consistent with our previous observations in fur seals sleeping on land [6]. In contrast to USWS, both eyes were closed during the rare episodes of REM sleep.

During the periods of rest in which the association between USWS and eye state was quantified in this study, both seals spent more time in R-USWS than in L-USWS. One of these seals (A, male) also spent more time resting in the lateral position on the left side than on the right side (S1 Table). When sleeping in the lateral posture, fur seals display USWS in the hemisphere which is ipsilateral to the water [7,10]. This would suggest that over the period of the study (11 days), this seal displayed greater amount of sleep in the right hemisphere compared to the left hemisphere regardless of its sleep posture. However, since the second fur seal (B, female) spent an equal amount of time resting in the lateral position on each side, the amount of time in the two hemisphere appears to be comparable in the second seal. We also reported that during any single day or even several days, the amount of R-USWS or L-USWS in cetaceans [1,3] or in fur seals [7,8] in one hemisphere can be greater than in the other hemisphere. However, whenever recordings were conducted for several continuous days and averaged, the difference between two hemispheres in the amount of USWS became less expressed or disappeared. Thus, the prevalence of R-USWS in these two fur seals during sleep in a prone position neither implies nor excludes behavioral asymmetry with regards to sleeping posture or hemispheric lateralization of USWS. This feature of sleep in fur seals merits specific attention in the future, if data are collected for a larger group of seals.

Our data shows that USWS in seals represents about 60% of rest in the prone position. In spite of the general correlation between the state of two eyes and unihemispheric sleep and waking in the fur seal (as described in cetaceans, [2,6]), the eye state alone is not reliable criteria to discriminate USWS and QW in seals resting in water in the sleep posture. The asymmetry in eye state also becomes apparent before slow waves appear in the EEG. On the contrary, REM sleep in the fur seal while in water can be reliably identified both in the prone and lateral positions based on behavioral features such as dropping and submerging the head into the water [10].

While our study examined a prone posture, fur seals more often sleep in water in the lateral sleep position. It has been suggested that this posture aids with thermoregulation, since 3 out of 4 flippers are held in the air, thus reducing heat loss in the cold water and the unilateral muscle activity of the 4^th^ flipper generates heat (e.g., [7,10–12,32,33]). Keeping its nostrils above the water, the fur seal can maintain a regular breathing pattern. In addition, this lateral position facilitates monitoring of the aquatic environment, since one eye is turned to or immersed in seawater. Although this eye is difficult to monitor, infrequent observations suggest that this eye periodically opens. Thus, we believe that while the prone posture may differ from the lateral posture with respect to movement and thermoregulation, the correlation of eye state and USWS in the prone posture is likely representative of the lateral sleep posture as well.

The fur seal has no terrestrial predators. However, animals of all sex and age groups can be harassed by territorial adult males during the breeding season [12]). The disturbance from conspecifics alone does not appear to be critical enough to require USWS in all pinnipeds while sleeping on the beach since it has not led to the evolution of USWS in the seals of the Phocidae family [34,35]. While in the ocean, fur seals are prey for transient killer whales and sharks approaching from the depth [12,13]. When in water, fur seals sleep at the surface. Both predators are silent while hunting, which might explain the need for continued visual vigilance in fur seals, despite the superiority of sound versus light transmission in water.

The seals in our study had lived in the facility for 1–2 years and were well adapted to captivity. Inherently, this laboratory experiment cannot incorporate the variable environmental conditions present in the wild, including the presence of predators, conspecifics, and anthropogenic disturbances. However, during sleep in the water the fur seals scanned the water environment continuously, despite the fact that there was no danger in the empty pool. In contrast to fur seals, phocids are deep divers. They have developed a different strategy to sleep in the water. In laboratory studies, phocids displayed sleep while holding their breath and submerging [34,35]. In the wild they appear to be capable of sleeping while performing deep dives or even hiding between rocks and at the bottom where they are better protected from predators and other disturbances [36,37]. However, hypotheses surrounding sleep in the wild are still to be tested.

We found that in the fur seal sleeping in seawater in the laboratory pool the eye contralateral to the waking hemisphere is continuously open for around a quarter of total USWS time. A quarter of total sleep time may seem to be a small portion. However, the duration of episodes of closure of the “waking” eye is usually much shorter than that of the eye contralateral to the “sleeping” hemisphere. Thus, instead of keeping the eye directed to water continuously open, the sleeping fur seal avoids closure of this eye for a period longer than several seconds. As a consequence, fur seals manage to achieve intermittent visual vigilance accounting for, on average, 40% of their sleep time in water. In addition, when in water, seals greatly reduce time spent in other types of sleep, during which the animal closes both eyes (such as bilateral SWS and REM, [7,10]). Therefore, having the eye directed to water intermittently open would presumably reduce the risk of predation.

The hypothesized ability of dolphins to have USWS during swimming suggested that the waking hemisphere facilitates motion [1] but no experimental evidence has been published to support this hypothesis. It was also hypothesized that birds may need USWS to sleep while in flight [38]. However, a recent study in great frigate birds did not show that USWS is required to maintain movement in flying birds. Instead, indirect evidence suggests that USWS may aid to control where the flying bird is circling in rising currents [21]. Compared to other studies, the fur seal data convincingly demonstrate that USWS facilitates both movement and visual vigilance. Thus, USWS serves these functions in the fur seal, which are likely to extend to cetaceans as well.

## Supporting information

S1 Table Rest in lateral and prone positions in fur seals in seawater. The amounts of rest and sleep recorded in four fur seals in seawater over the entire study period.
(DOCX)

S2 Table Characteristics of episodes of rest and sleep in fur seals in a prone position. The amounts of QW, USWS and REM sleep recorded in two fur seals during all rest episodes.
(DOCX)

S3 Table Characteristics of the state of the eye contralateral to the waking hemisphere during unihemispheric sleep in fur seals. The duration of opening and closure episodes of the eye contralateral to the waking hemisphere (L or R eye depending on where sleep is occurring) during all USWS episodes in two fur seals.
(DOCX)

S1 File EEG power in the left and right cerebral hemispheres and the left and right eye state in fur seals resting in a prone position. Data for all rest and sleep episodes recorded in two fur seals and the results of statistical tests.
(XLSX)
