# Supplementary material for: Eye state asymmetry during aquatic unihemispheric slow wave sleep in northern fur seals (Callorhinus ursinus)
Source: PLoS One. 2019 May 22;14(5):e0217025. doi: 10.1371/journal.pone.0217025 (PMC6530852; doi:10.1371/journal.pone.0217025)
Supplement: S1 Table — The amounts of rest and sleep recorded in four fur seals in seawater over the entire study period. (DOCX) [file pone.0217025.s001.docx]

**S1 Table. Rest in lateral and prone positions in fur seals in seawater.**

|  | Seal A | Seal B | Seal C | Seal D | Sum |
| --- | --- | --- | --- | --- | --- |
| Number of experimental days during which the behavioral (video) data were collected ^a^ | 11 | 13 | 11 | 10 | 45 |
|  |  |  |  |  |  |
| Amount of REST in ^b^  (min) | Seal A | Seal B | Seal C | Seal D | - |
| Lateral position, R-side | 609 | 1058 | 568 | 1329 | - |
| Lateral position, L-side | 889 | 1066 | 873 | 1265 | - |
| Prone position | 342 | 1158 | 0 | 0 | - |
| Lateral position, R+L sides | 1498 | 2124 | 1442 | 2593 | - |
| All positons | 1841 | 3282 | 1442 | 2593 |  |
|  |  |  |  |  |  |
| Amount of REST in ^c^  (% of total rest time) | Seal A | Seal B | Seal C | Seal D | Mean + SEM  (n=4) |
| Prone position | 19% | 35% | 0% | 0% | 13+8% |
| Lateral position, R+L sides | 81% | 65% | 100% | 100% | 87+8% |
| REST in the lateral position on the right side (% rest on both sides) | 41% | 50% | 39% | 51% | 45+3% |
|  |  |  |  |  |  |
| Amount of REST in ^d^  (% of 24-h) | Seal A | Seal B | Seal C | Seal D | Mean + SEM  (n=4) |
| Lateral position, R-side | 11.5% | 17.0% | 10.8% | 27.7% | 16.7+3.9% |
| Lateral position, L-side | 16.8% | 17.1% | 16.5% | 26.3% | 19.2+2.4% |
| Prone position | 6.4% | 18.6% | 0.0% | 0.0% | 6.2+4.4% |
| Lateral position, R+L sides | 28.4% | 34.0% | 27.3% | 54.0% | 35.9+6.2% |
| Total Rest Time | 34.8% | 52.6% | 27.3% | 54.0% | 42.2+6.6% |
|  |  |  |  |  |  |
| Number of experimental days during which the data were collected | Seal A | Seal B | Seal C | Seal D | Sum |
| Behavioral and electrophysiological | 10 | 6 | 6 | 10 | 32 |
| Behavioral and electrophysiological,  and the seal rested in a prone position | 4 | 1 | 0 | 0 | 5 |
| Amount of SLEEP that occurred in a prone position (min) ^e^ | 424 | 72 |  |  |  |
| Amount of SLEEP in a prone position analyzed (min) ^f^ | 337 | 51 |  |  |  |
| Total SLEEP time analyzed (%) | 80% | 71% |  |  |  |

The amounts of rest and sleep recorded in four fur seals in seawater over the entire study period.

^a^ Behavior of 4 fur seals (A-D) was scored in 45 complete days (24 hours) of 46 days during which the seals were in seawater. For 1 day, the recording was not conducted due to technical problems.

^b^ Amount of REST in min for the entire period of recording in each seal (shown above).

^c^ Amount of REST in the lateral and prone positions as a percent of the total amount of rest or amount of rest in the lateral position on both sides for the entire period of recording in each seal (shown above) and mean + standard error for all 4 seals.

^d^ Amount of REST as a percent of 24-h for each seal and mean + standard error for all 4 seals.

^e^ Amount of sleep which occurred in the prone position during 4 days for seal A and 1 day for seal B.

^f^ Amount of sleep for which the association between USWS and eye state was examined.
