# Supplementary material for: Eye state asymmetry during aquatic unihemispheric slow wave sleep in northern fur seals (Callorhinus ursinus)
Source: PLoS One. 2019 May 22;14(5):e0217025. doi: 10.1371/journal.pone.0217025 (PMC6530852; doi:10.1371/journal.pone.0217025)
Supplement: S2 Table — The amounts of QW, USWS and REM sleep recorded in two fur seals during all rest episodes. (DOCX) [file pone.0217025.s002.docx]

**S2 Table. Characteristics of episodes of rest and sleep in fur seals in a prone position.**

| Rest episode  (number and type) | Seal | Date | Time of day | Rest | QW | L-USWS  (min) | R-USWS | REM |
| --- | --- | --- | --- | --- | --- | --- | --- | --- |
| 1, R-USWS | A | 29-May | 09:58-13:01 | 182.7 | 70.3 | 0.0 | 111.3 | 1.1 |
| 2, R-USWS | A | 31-May | 09:50-13:45 | 235.0 | 111.0 | 0.0 | 124.0 | 0.0 |
| 3, R-USWS & L-USWS | A | 2-Jun | 00:21-03:03 | 162.0 | 82.3 | 32.3 | 43.7 | 3.7 |
| 4, L-USWS | A | 4-Jun | 01:10-01:51 | 41.0 | 20.0 | 18.3 | 0.0 | 2.7 |
| 5, L-USWS | B | 1-Jul | 20:48-21:20 | 30.7 | 16.7 | 14.0 | 0.0 | 0.0 |
| 6, R-USWS | B | 2-Jul | 03:12-04:10 | 57.7 | 20.3 | 0.0 | 34.7 | 2.7 |
| Total (min) |  |  |  | 709.1 | 320.6 | 64.6 | 313.7 | 10.2 |
| % of TRT |  |  |  | 100.0% | 45.2% | 9.1% | 44.2% | 1.4% |

The amounts of QW, USWS and REM sleep recorded in two fur seals during all rest episodes. A period of rest was defined as an episode which started when the seal took the prone posture and ended when the seal resumed active behavior (swimming or grooming). R-USWS and L-USWS, USWS in the right and left hemisphere, respectively. QW, quiet wakefulness. REM, rapid eye movement sleep. TRT, total recording time. Min, minutes. This table includes information on all 6 episodes of rest in the prone position recorded in seal A and B. The rest episodes are numbered in column 1. The type of USWS (R-USWS, L-USWS or both) is listed after the comma.
