# Supplementary material for: Eye state asymmetry during aquatic unihemispheric slow wave sleep in northern fur seals (Callorhinus ursinus)
Source: PLoS One. 2019 May 22;14(5):e0217025. doi: 10.1371/journal.pone.0217025 (PMC6530852; doi:10.1371/journal.pone.0217025)
Supplement: S3 Table — The duration of opening and closure episodes of the eye contralateral to the waking hemisphere (L or R eye depending on where sleep is occurring) during all USWS episodes in two fur seals. (DOCX) [file pone.0217025.s003.docx]

**S3 Table. Characteristics of the state of the eye contralateral to the waking hemisphere during unihemispheric sleep in fur seals.**

| Number of episodes when this eye was closed | | | | | | | |  |  |  |  |
| --- | --- | --- | --- | --- | --- | --- | --- | --- | --- | --- | --- |
| Episodes ^a^  duration | A1-R | A2-R | A3.1-R | A3.2-L | A4-L | B1-L | B2-R | Mean | SEM | Min | Max |
| 1 sec | 174 | 223 | 15 | 42 | 3 | 39 | 10 | 72.3 | 33.5 | 3 | 223 |
| 2 sec | 178 | 221 | 23 | 71 | 4 | 52 | 8 | 79.6 | 32.6 | 4 | 221 |
| 3 sec | 175 | 146 | 40 | 68 | 1 | 30 | 8 | 66.9 | 25.7 | 1 | 175 |
|  |  |  |  |  |  |  |  |  |  |  |  |
| Amount of time when this eye was closed | | | | | | | |  |  |  |  |
| Episodes ^a^  duration | A1-R | A2-R | A3.1-R | A3.2-L | A4-L | B1-L | B2-R | Mean | SEM | Min | Max |
| 1 sec | 174 | 223 | 15 | 42 | 3 | 39 | 10 | 72.3 | 33.5 | 3 | 223 |
| 2 sec | 530 | 665 | 61 | 184 | 11 | 143 | 26 | 231.4 | 98.4 | 11 | 665 |
| 3 sec | 1055 | 1103 | 181 | 388 | 14 | 233 | 50 | 432.0 | 173.4 | 14 | 1103 |
|  |  |  |  |  |  |  |  |  |  |  |  |
| The total duration of the open and closed state of this eye | | | | | | | |  |  |  |  |
| Episodes ^a^  Eye state | A1-R | A2-R | A3.1-R | A3.2-L | A4-L | B1-L | B2-R | Mean | SEM | Min | Max |
| Open (sec) | 1576 | 1807 | 254 | 614 | 198 | 293 | 142 | 72.3 | 33.5 | 3 | 223 |
| Open or closed, TRT^2^ (sec) | 6517 | 7270 | 1223 | 1921 | 1090 | 835 | 763 | 231.4 | 98.4 | 11 | 665 |
| Open (% of TRT) | 24.2 | 24.9 | 20.8 | 32.0 | 18.2 | 35.1 | 18.6 | 432.0 | 173.4 | 14 | 1103 |
|  |  |  |  |  |  |  |  |  |  |  |  |
| Amount of time this eye was closed (% of TRT) | | | | | | | |  |  |  |  |
| Episodes ^a^  duration | A1-R | A2-R | A3.1-R | A3.2-L | A4-L | B1-L | B2-R | Mean | SEM | Min | Max |
| 1 sec | 2.7 | 3.1 | 1.2 | 2.2 | 0.3 | 4.7 | 1.3 | 2.1 | 0.5 | 0 | 5 |
| 2 sec | 8.1 | 9.1 | 5.0 | 9.6 | 1.0 | 17.1 | 3.4 | 7.5 | 2.0 | 1 | 17 |
| 3 sec | 16.2 | 15.2 | 14.8 | 20.2 | 1.3 | 27.9 | 6.6 | 14.3 | 3.3 | 1 | 28 |
|  |  |  |  |  |  |  |  |  |  |  |  |
| Amount of time this eye was not closed (% of TRT) longer than | | | | | | | |  |  |  |  |
| Episodes ^a^  duration | A1-R | A2-R | A3.1-R | A3.2-L | A4-L | B1-L | B2-R | Mean | SEM | Min | Max |
| 1 sec | 26.9 | 27.9 | 22.0 | 34.1 | 18.4 | 39.8 | 19.9 | 27.0^3^ | 2.9 | 18 | 40 |
| 2 sec | 32.3 | 34.0 | 25.8 | 41.5 | 19.2 | 52.2 | 22.0 | 32.5 | 4.4 | 19 | 52 |
| 3 sec | 40.4 | 40.0 | 35.6 | 52.2 | 19.4 | 63.0 | 25.2 | 39.2 | 5.6 | 19 | 63 |

The duration of opening and closure episodes of the eye contralateral to the waking hemisphere (L or R eye depending on where sleep is occurring) during all USWS episodes in two fur seals.

^a^ Episodes: the letter is seal A or B; the digit is the episode number; R and L is USWS in the right (R) or left (L) hemispheres.

^b^ TRT is the total recording time. SEM – standard error. Min and Max – minimal and maximal values.
